# Clinical frailty assessment might be associated with mortality in incident dialysis patients

**DOI:** 10.1038/s41598-022-22483-8

**Published:** 2022-10-21

**Authors:** Rikako Oki, Yoshifumi Hamasaki, Shiho Tsuji, Kana Suzuki, Sayaka Tsuneishi, Mikie Imafuku, Yohei Komaru, Yoshihisa Miyamoto, Ryo Matsuura, Kent Doi, Masaomi Nangaku

**Affiliations:** 1 Department of Hemodialysis and Apheresis, The University of Tokyo Hospital, 7-3-1 Hongo, Bunkyo-ku, Tokyo, 113-8655 Japan; 2 Department of Acute Medicine, The University of Tokyo Hospital, Tokyo, Japan

**Keywords:** Haemodialysis, Risk factors

## Abstract

Frailty is associated with mortality in maintenance dialysis patients. For incident dialysis patients, we used the clinical frailty scale (CFS) to investigate frailty as related to mortality or hospitalization within 2 years. We retrospectively reviewed the medical records of patients initiating hemodialysis or peritoneal dialysis during 2016–2018. Based on those records, two dialysis nurses independently used a 9-point CFS (1 = “Very fit” to 9 = “Terminally ill”) to assess each patient’s frailty at dialysis initiation. Patients with a mean CFS value of 5 or higher were classified into the frail group. The 2-year survival rates or hospitalization-free rates after the initiation of dialysis were compared between the frail (mean CFS score ≥ 5) and non-frail (mean CFS score < 5) groups. The analysis included 155 incident dialysis patients with mean age of 66.7 ± 14.1 (71% male). Frailty was inferred for 39 (25%) patients at dialysis initiation. Kaplan–Meier analyses showed that the survival rate and hospitalization-free rate within 2 years were significantly lower in the frail group than in the non-frail group (*p* < 0.01). Cox proportional hazards regression analyses revealed the CFS score as associated with the occurrence of a composite outcome, independently of age (hazard ratio 1.34, 95% confidence interval 1.04–1.72). Frailty assessment based on clinical judgment using CFS might predict adverse outcomes in dialysis-initiated patients.

## Introduction

Frailty is recognized as a state of loss of reserves (energy, physical ability, cognition, and health) that engenders increased vulnerability^1^. Even minor stress in patients with frailty can degrade health and make it difficult to maintain life functions because of aging-related decline in the reserve capacities of physical and psychological functions. Frail individuals deserve special attention because they experience adverse health outcomes at high rates^2^.

Difficulties presented by frailty, which are discussed often nowadays, affect dialysis patients. Earlier studies have revealed high prevalence of frailty not only in older dialysis patients, but also in younger ones^3^. For maintenance dialysis patients, frailty is reportedly an independent risk factor for poor outcomes such as mortality, hospitalization, falls, and discharge to an assisted care facility^4,5^. According to a retrospective study analyzing data from the Nationwide Inpatient Sample, hospitalized maintenance dialysis patients with frailty have twice the risk of in-hospital mortality and three times the risk of discharge to long-term facilities. Moreover, they require higher in-hospital medical costs than non-frail dialysis patients^6^. Frailty severely affects the health not only of maintenance dialysis patients, but also of incident dialysis patients^7–9^. Fitzpatrick et al. reported that frailty in incident hemodialysis patients is associated with a 1.66-fold elevated mortality risk, even among patients with abdominal obesity^10^.

Various tools have been developed for frailty assessment. They are being used for clinical practice and research. Fried’s criteria are the most commonly used. Frailty is diagnosed when three or more of the five major physical components of frailty are identified: unintentional weight loss, low physical activity level, weakness (low grip strength), exhaustion, and slowness (slow gait speed)^11^. However, these criteria, which require equipment for physical measurements, are unsuitable for detailed assessment of the degree of frailty.

The Clinical Frailty Scale (CFS) developed by Rockwood et al. is an assessment tool that enables assessment of the degree of frailty based on clinical judgment^1^. The original version of CFS was a 7-point scale with grading of the degree of frailty from 1 (very fit) to 7 (severely frail). In 2020, an updated version of CFS (CFS ver. 2.0) was introduced with revised level names. It was edited to level descriptions by 9 scales ranging from 1 (very fit) to 9 (terminally ill)^12^. Using CFS, health-care professionals assess the level of frailty based on clinical information about patient activities of daily living such as mobility, dressing, and preparing meals. Silhouettes and descriptions of a typical patient in each CFS category are helpful for the proper classification of patients. For the screening of frailty, CFS is recognized as a reliable tool. Earlier reports have suggested that CFS is a predictor of mortality or adverse outcomes in people with various diseases^13,14^. However, because few studies have examined incident dialysis patients, it has not been well established whether the degree of frailty evaluated by CFS at dialysis initiation is associated with short-term adverse outcomes. In addition, only the 7-point CFS scale, not the 9-point version, was used for those earlier studies^7,8^. The aim of this study is investigation of the relation between frailty as assessed by CFS on a 9-point scale at the time of dialysis initiation and adverse outcomes such as death or hospitalization within 2 years.

## Materials and methods

### Patients and data collection

We conducted a retrospective cohort study of adult patients who had been admitted to a tertiary care hospital and who had started chronic dialysis (either peritoneal dialysis or hemodialysis) as their first kidney replacement therapy between January 1, 2016 and December 31, 2018. In our clinical practice, all patients start dialysis under inpatient care. After discharge, they go to dialysis clinics near their home. This study, which was conducted in accordance with the principles outlined in the Declaration of Helsinki, was approved by the Institutional Review Board of The University of Tokyo (#2269). Written informed consent was obtained from all patients at dialysis initiation. Adult (age 18 years and older) incident dialysis patients who started chronic dialysis as their first kidney replacement therapy in our hospital between January 1, 2016 and December 31, 2018 were included in this study. The exclusion criteria were the following: (1) patients lost to follow-up after discharge; (2) patients who only received dialysis transiently (< 1 week); and (3) patients who had been hospitalized for more than 3 months before dialysis initiation (because long hospitalization might affect the degree of frailty). In addition, (4) one patient died suddenly during his first hemodialysis session.

Basic information of the patients was obtained from their medical records: age, gender, cause of end-stage renal disease, history of cardiovascular disease (CVD), and days of hospitalization in initiation of dialysis. Clinical (systolic and diastolic blood pressure, body mass index) and laboratory data from the time of dialysis initiation were also obtained from medical records: hemoglobin (Hb), blood urea nitrogen (BUN), serum creatinine (Cr), estimated glomerular filtration rate (eGFR), corrected calcium (cCa), phosphate (IP), total protein (TP), albumin (Alb), C-reactive protein (CRP), uric acid (UA), hemoglobin A1c (HbA1C), total cholesterol (T.chol), triglyceride (TG), and brain natriuretic peptide (BNP).

### Evaluation of CFS

To assess and quantify the level of frailty, CFS ver. 2.0, graded on a 1–9 scale, was used: 1, very fit; 2, fit; 3, managing well; 4, living with very mild frailty; 5, living with mild frailty; 6, living with moderate frailty; 7, living with severe frailty; 8, living with very severe frailty; 9, terminally ill^12^. We applied to the licensor for the use of CFS ver. 2.0 translated into Japanese and received their kind permission. Two dialysis nurses, one with more than 10 years of nursing experience and the other with more than 2 years of working at our dialysis center, independently assessed frailty using CFS by reference to medical chart records on activities of daily living at the time of dialysis initiation. The mean value of the results of the two nurses’ assessments was defined as the patient’s CFS score. Patients with a mean CFS score of 5 or higher were classified into the frail group; the remainder were classified into the non-frail group.

### Outcomes

The primary outcome was the composite of all-cause mortality and hospitalization within 2 years after dialysis initiation. The secondary outcome was all-cause mortality within 2 years after dialysis initiation. The occurrence and date of the first observed outcomes after the start of dialysis were investigated. A researcher other than the two nurses evaluating CFS was responsible for collecting prognostic data through medical records and telephone surveys. Patient survival was censored at kidney transplantation or the date of last follow-up.

### Statistical analysis

All statistical analyses were conducted using software (JMP^®^, Ver. < 16.0 > ; SAS Institute Inc., Cary, NC, 1989–2021). Continuous data were expressed as mean ± standard deviation or median (interquartile range). Student *t*-tests or Mann–Whitney U-tests were used to compare continuous variables. The chi-square test or Fisher’s exact test was used to compare categorical variables. The Kaplan–Meier method and the log-rank test were used to compare differences in primary and secondary outcomes between the frail (mean CFS ≥ 5) and non-frail (mean CFS < 5) groups. Univariate and multivariate Cox proportional hazards regression analyses were performed to examine significant factors associated with the primary outcome. A Spearman’s correlation test was performed on correlation between the CFS score and clinical factors. Weighted Cohen’s kappa and the intraclass correlation coefficient (ICC) were calculated to quantify inter-rater and intra-rater reliability. Values for which *p* was less than 0.05 were inferred as significant.

### Ethical approval

This study was conducted in accordance with the principles outlined in the Declaration of Helsinki and was approved by the Institutional Review Board of The University of Tokyo (#2269).

## Results

### Characteristics of study participants

**Figure 1:**
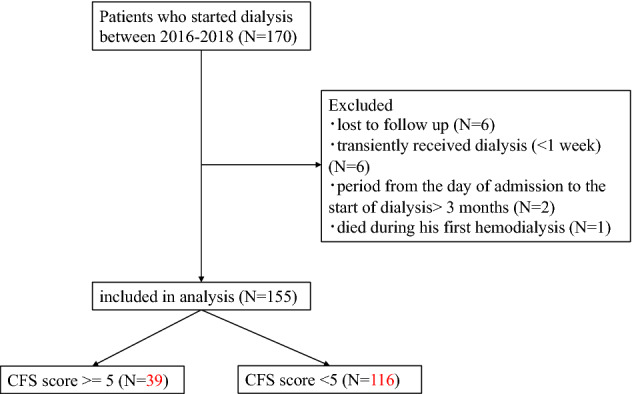
Chart showing flow of the study.

Of the 170 patients who started dialysis during the study period, a total of 155 patients were included in the analysis (Fig. 1). Baseline characteristics and laboratory data of all subjects are presented in Table 1. The mean patient age was 66.7 ± 14.1 years old and 110 (71%) were male. The overall 2-year mortality was 17% (27 deaths). The causes of death included malignancy (*n* = 6, 22%), CVD (*n* = 5, 18%), infection (*n* = 4, 15%), others (*n* = 5, 19%), and unknown (*n* = 7, 26%). The causes of unexpected hospitalization (*n* = 53) included CVD (*n* = 16, 30%), infection (*n* = 14, 26%), vascular access troubles (*n* = 4, 8%), malignancy (*n* = 8, 15%), and others (*n* = 11, 21%). The most common primary kidney disease was diabetic kidney disease (*n* = 61, 39%), followed by chronic glomerulonephritis (*n* = 31, 20%) and nephrosclerosis (*n* = 24, 16%). Among all 155 patients, the number of patients with mean CFS ≥ 5 (frail group) was 39 (25%).

**Table 1:** Comparison between groups with mean CFS scores of more or less than 5.

Variables	All (*N* = 155)	CFS ≥ 5 (*N* = 39)	CFS < 5 (*N* = 116)	*p*
Age (y.o.)	66.7 ± 14.1	68.7 ± 17.8	66.0 ± 12.7	0.30
Male (*n *(%))	110 (71%)	28 (72%)	82 (71%)	0.90
**Primary kidney disease**				0.29
Chronic glomerulonephritis	31 (20%)	5 (13%)	26 (23%)	
Diabetic kidney disease	61 (39%)	19 (49%)	42 (36%)	
Nephrosclerosis	24 (16%)	4 (10%)	20 (17%)	
Others	39 (25%)	11 (28%)	28 (24%)	
Planned initiation of dialysis	92 (59%)	9 (23%)	83 (72%)	< 0.01
Hemodialysis/peritoneal dialysis	138/17	39/0	99/17	< 0.01
Hospitalization period (days)	12 (7, 30)	31 (15, 55)	10 (6, 22)	< 0.01
2-year mortality (*n *(%))	27 (17%)	16 (41%)	11 (9%)	< 0.01
Hospitalization or death in 2 years	69 (45%)	27 (69%)	42 (36%)	< 0.01
History of CVD (*n *(%))	66 (43%)	21 (54%)	45 (39%)	0.10
Systolic blood pressure (mmHg)	149.6 ± 21.6	146.9 ± 28.5	150.5 ± 18.9	0.38
Diastolic blood pressure (mmHg)	76.6 ± 15.8	78.2 ± 21.8	76.1 ± 13.2	0.48
Body mass index (kg/m^2^)	23.9 (21.0, 27.6)	24.1 (21.8, 28.6)	23.6 (20.7, 27.4)	0.21
Hb (g/dl)	9.09 ± 1.60	8.99 ± 1.96	9.12 ± 1.48	0.65
Cr (mg/dl)	8.49 (6.90, 10.5)	7.49 (5.93, 9.45)	8.75 (7.20, 10.7)	<0 .01
BUN (mg/dl)	90.2 (73.5, 106)	95.5(74.6, 119.2)	89.4(72.7,101.3)	0.22
eGFR (ml/min/1.73 m^2^)	5.25 (4.09, 6.32)	5.96 (4.51, 7.69)	5.05 (4.07, 6.07)	< 0.01
Corrected Ca (mg/dl)	8.80 (8.40, 9.10)	8.80 (8.30, 9.20)	8.70 (8.40, 9.10)	0.84
IP (mg/dl)	5.60 (4.8, 6.70)	5.50 (4.70, 7.22)	5.70 (4.80, 6.58)	0.59
TP (g/dl)	6.03 ± 0.71	5.71 ± 0.89	6.13 ± 0.61	< 0.01
Alb (g/dl)	3.13 ± 0.55	2.78 ± 0.59	3.25 ± 0.48	< 0.01
CRP (mg/dl)	0.20 (0.06, 1.11)	1.04 (0.12, 5.66)	0.16 (0.05, 0.52)	< 0.01
T.chol (mg/dl)	157 (127, 183)	136 (112, 187)	157 (135, 183)	0.18
TG (mg/dl)	118 (89.5, 163)	113 (84.0, 159)	120 (91, 170)	0.25
UA (mg/dl)	7.20 (6.10, 8.80)	8.10 (6.10, 9.60)	7.15 (6.03, 8.30)	0.09
HbA1C (%)	5.70 (5.30, 6.20)	5.75 (5.23, 6.48)	5.60 (5.30, 6.03)	0.38
BNP (pg/ml)	206 (72.7, 688)	544 (222, 1377)	165 (63.0, 408)	< 0.01

Continuous data are presented as mean ± SD or median (IQR): *CVD* cardiovascular disease, *Hb* hemoglobin, *Cr* creatinine, *BUN* blood urea nitrogen, *Egfr* estimated glomerular filtration rate, *Ca* calcium, *IP* phosphate, *TP* total protein, *Alb* albumin, *CRP* C-reactive protein, *T.chol* total cholesterol, *TG* triglyceride, *UA* uric acid, *HbA1C* hemoglobinA1C, *BNP* brain natriuretic peptide.

Table 1 presents results of comparison between the two groups with mean CFS scores greater than or equal to 5, and less than 5. The group with mean CFS ≥ 5 (frail group) had higher 2-year mortality (41% vs. 9%, *p* < 0.01), hemodialysis patients, (100% vs. 85%, *p* < 0.01), CRP [1.04 (0.12, 5.66) vs. 0.16 (0.05, 0.52) mg/dl, *p* < 0.01], and BNP [544 (222, 1377) vs. 165 (63.0, 408) pg/ml, *p* < 0.01] than patients with CFS < 5 (non-frail group). The serum creatinine, total protein, and albumin concentrations were significantly lower in the frail group. More patients in the frail group started dialysis urgently (planned initiation of dialysis; 23% vs. 72%, *p* < 0.01) and had longer hospitalization periods (31 days vs. 10 days, *p* < 0.01) than patients of the non-frail group.

### Validity of CFS scores

**Figure 2:**
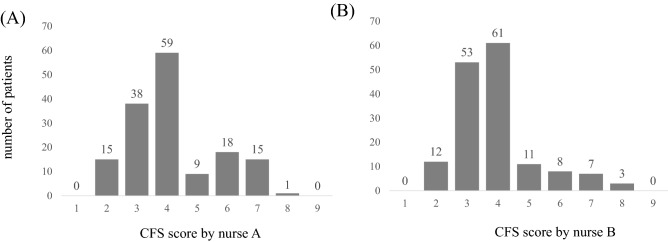
Distribution of CFS scores by two nurses. The category of CFS scores divided by 5 were 86% identical between two nurses.

The distribution of the CFS results assessed by each of the two nurses is presented in Fig. 2. The mean value of the CFS scores reported by the two nurses was 4.03 ± 1.25; 25% (*N* = 39) for the patients were evaluated as mildly to severe frailty (mean CFS ≥ 5). Agreement between the two nurses' evaluation of whether the CFS score was more than 5 (living with mild to very severe frailty) or not was found in 133 (86%) of all cases.

The inter-rater reliability between two dialysis nurses for determining the presence of frailty (CFS ≥ 5 or not) was assessed using a weighted Cohen’s kappa of 0.64, indicating fair to good agreement^15^. In addition, the intraclass correlation coefficient (ICC) for CFS scoring was calculated as 0.80, indicating good reliability^16^.

Table 2 displays the correlation found between mean CFS score obtained from clinical judgement and objective parameters. Significant positive correlation was found between the mean CFS score and the hospitalization period (*r* = 0.43, *p* < 0.01), CRP (*r* = 0.41, *p* < 0.01), UA (*r* = 0.25, *p* < 0.01), and BNP (*r* = 0.33, *p* < 0.01). Significant negative correlation was found between the mean CFS score and Cr (*r* = − 0.23, *p* < 0.01) TP (*r* = − 0.20, *p* < 0.01), and Alb (*r* = − 0.43, *p* < 0.01).

**Table 2:** Correlation between mean CFS scores obtained by subjective assessment and objective parameters.

Parameters	*r*	*p*
Age	0.16	0.04
Hospitalization period	0.43	< 0.01
Systolic blood pressure	− 0.11	0.17
Diastolic blood pressure	− 0.03	0.69
Body mass index	0.13	0.11
Hb	− 0.15	0.055
Cr	− 0.23	< 0.01
BUN	0.16	0.052
eGFR	0.26	< 0.01
Corrected Ca	− 0.01	0.89
IP	0.19	0.02
TP	− 0.20	0.01
Alb	− 0.43	< 0.01
CRP	0.41	< 0.01
T.chol	− 0.15	0.07
TG	− 0.12	0.16
UA	0.25	< 0.01
HbA1C	0.17	0.04
BNP	0.33	< 0.01

### Relation between outcomes and CFS

**Figure 3:**
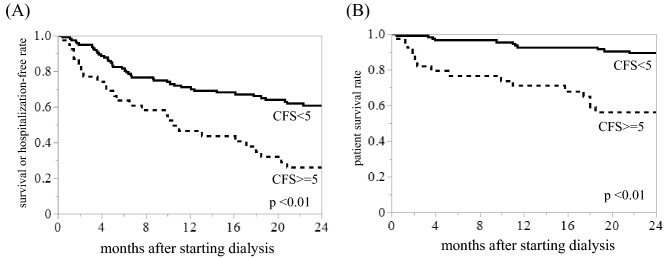
Kaplan–Meier analysis for the 2-year survival rate or probability of avoidance of the rehospitalization within 2 years after initiating dialysis. Composite outcomes of the survival rate or probability of avoidance of the rehospitalization (**A**) and survival rate (**B**) during 2 years from initiation of dialysis were significantly lower in the CFS ≥ 5 group than in the CFS < 5 group (*p* < 0.01, both).

The 2-year survival rate or hospitalization after the initiation of dialysis was compared between the frailty and non-frail groups using Kaplan–Meier analysis and log-rank testing (Fig. 3A). Results showed that worse frailty (mean CFS score ≥ 5) was associated with decreased 2-year survival or hospitalization-free rate within 2 years (*p* < 0.01, Fig. 3A). Similarly, the 2-year survival rate in CFS ≥ 5 group was significantly lower than that in CFS < 5 group (*p* < 0.01, Fig. 3B). Based on Kaplan–Meier analysis results, significant difference in the 2-year survival rate or hospitalization after the initiation of dialysis was inferred when cutoff of the mean CFS was set as 4 (*p* = 0.03, supplementary Fig. 1A). The 2-year survival rate was significantly lower in the CFS ≥ 4 group than in the CFS < 4 group (*p* < 0.01, supplementary Fig. 1B). Similar results were obtained when setting the cut-off of mean CFS to 6 (Supplementary Fig. 2AB).

Cox proportional hazard models were used to evaluate the relation between 2-year mortality or hospitalization and mean CFS score. The analyses were adjusted for factors that were reported as associated with an increased risk of all-cause mortality: age, expected start of dialysis, systolic blood pressure, Cr, Alb, CRP, total cholesterol, and BNP (Tables 3, 4)^17–21^. Increase in the mean CFS score was found to be related significantly to 2-year mortality or hospitalization in all models (Table 4). Similarly, Cox regression analysis suggested that an increase in the mean CFS score was associated with death within 2 years from the initiation of dialysis (Supplementary Tables 1 and 2). Adjustment for age and planned initiation of dialysis revealed a nearly twofold higher risk of 2-year mortality for each 1-point increase in the mean CFS. The hazard ratio was also considerably higher when adjusted for each factor above (Supplementary Table 2).

**Table 3:** Results of univariate cox proportional hazards model analyses for 2-year mortality or hospitalization.

Variables	HR (95% CI)	*p*
Age	1.01 (0.99–1.03)	0.22
Male	1.35 (0.78–2.33)	0.27
Diabetic kidney disease as primary kidney disease	1.16 (0.72–1.88)	0.53
Planned initiation of dialysis	0.65 (0.40–1.04)	0.07
Hemodialysis (vs. peritoneal dialysis)	0.91 (0.55–2.22)	0.78
Hospitalization period	1.01 (1.00–1.02)	< 0.01
History of CVD	1.15 (0.72–1.85)	0.56
Systolic blood pressure	0.98 (0.97–0.99)	< 0.01
Diastolic blood pressure	0.996 (0.98–1.01)	0.63
Body mass index	1.01 (0.97–1.05)	0.60
Mean CFS score	1.46 (1.22–1.73)	< 0.01
Hb	0.91 (0.78–1.07)	0.27
Cr	0.86 (0.78–0.95)	< 0.01
BUN	1.00 (0.99–1.01)	0.94
eGFR	1.15 (1.04–1.25)	< 0.01
Corrected Ca	0.83 (0.61–1.14)	0.26
IP	0.95 (0.80–1.11)	0.56
TP	0.96 (0.67–1.37)	0.82
Alb	0.63 (0.41–0.98)	0.04
CRP	1.08 (1.02–1.14)	< 0.01
T.chol	0.99 (0.987–0.998)	0.01
TG	1.00 (0.99–1.01)	0.10
UA	1.06 (0.93–1.20)	0.40
HbA1C	0.98 (0.72–1.29)	0.89
BNP	1.00 (0.99–1.00)	0.20

**Table 4:** Results from multivariate cox proportional hazards model for 2-year mortality or hospitalization.

Variables	Model 1		Mode1 2		Model 3	
	OR (95% CI)	*p*	OR (95% CI)	*p*	OR (95% CI)	*p*
Age	0.99 (0.98–1.01)	0.54	1.00 (0.98–1.02)	0.94	1.00 (0.98–1.02)	0.97
Mean CFS score	1.28 (1.00–1.61)	0.047	1.31 (1.02–1.67)	0.03	1.34 (1.04–1.72)	0.02
Planned initiation of dialysis	1.15 (0.61–2.15)	0.67	1.27 (0.68–2.38)	0.38	1.29 (0.68–2.43)	0.43
Systolic blood pressure	0.99 (0.98–1.00)	0.07	0.98 (0.97–0.996)	0.01	0.99 (0.97–0.998)	0.02
T chol	0.99 (0.99–1.00)	0.06	0.995 (0.99–1.00)	0.12	0.995 (0.99–1.00)	0.13
BNP	1.00 (0.99–1.00)	0.57	1.00 (0.99–1.00)	0.51	1.00 (0.98–1.02)	0.54
Cr	0.89 (0.780–0.99)	0.04				
Alb			0.76 (0.46–1.27)	0.29		
CRP					1.01 (0.92–1.09)	0.78

## Discussion

Results of this study demonstrated that frailty at the initiation of dialysis was associated with mortality or hospitalization within 2 years. This report is the first to describe a study demonstrating the predictive validity of 9-point CFS assessed by nurses for short-term prognosis in incident dialysis patients. We also found correlation between the CFS score based on the nurses’ assessment and several objective laboratory parameters such as CRP, Alb, Cr, and BNP.

In our study, 25% of the patients were classified into the frail group when judged by the criterion of mean CFS score ≥ 5. Clark et al. compared various frailty measures for assessments of incident dialysis patients. When the cutoff value of mean CFS was set as 5, CFS ≥ 5 correspondent frailty defined the frailty index with the highest specificity^22^. In an earlier report of 9-scale CFS and 30-day mortality, patients who were assigned a score of 5 or higher were regarded as frail^23^. Consequently, a mean CFS score ≥ 5 is a reasonable definition of frailty for our study.

The frequency of frailty in dialysis patients varies among reports. It seems to depend on the assessment method and patient background. Chu et al. demonstrated that frailty as assessed by Physical Frailty Phenotype (Fried’s criteria) was present in 71.4% among older incident hemodialysis patients and was present in 47.3% of younger ones^24^. Alfaadhel et al. reported frailty as assessed using 7-point CFS existed in 26% of all incident hemodialysis patients^7^, which result was compatible with our findings.

Screening for frailty is important because frailty is associated with increased risk of hospitalization, mortality, and falls in individuals with chronic kidney disease^25^. Increased frailty consequently engenders huge physical and mental burdens on patients. Alfaadhel et al. demonstrated that a higher CFS score at dialysis initiation was associated with higher mortality. Our findings are compatible with their results^7^. Our findings indicate that CFS, which can be assessed easily, might be a useful predictor of mortality in incident dialysis patients.

Although CFS is an assessment tool for frailty based on a health-care provider’s judgment, it has been shown to have high inter-rater reliability^1^. For this study, the categorization (CFS score is above 5 or not) was consistent in 133 (86%) cases. The 9-point CFS was apparently reliable with small inter-rater differences in the clinical assessment of incident dialysis patients. Additionally, we calculated and used the mean of CFS scores from the two nurses for analyses in this study. The mean of the CFS scores from two nurses, who care for patients under consideration of their conditions and daily lives, might increase the reliability of the results.

Correlation between CFS scores based on the clinician’s judgement and on various objective markers such as Alb, CRP, and BNP might support the reliability of CFS in our cohort. Serum albumin, known as an independent risk factor of mortality as well as a nutritional marker in end-stage renal disease (ESRD) patients, has been reported as lower in frail individuals than in healthy ones^17,26^. The mean serum albumin concentration was low, 3.14 ± 0.55 g/dl, which might reflect the patient condition, such as chronic malnutrition and exhaustive illness in ESRD patients. Frail patients often have comorbidities that can affect the inflammatory parameters^27^. Aggravation of inflammation can engender structural damage to physiologic organs such as musculoskeletal, hematological (anemia), cardiovascular, and endocrine systems^28^. Consequently, it is not surprising that serum CRP was correlated significantly with the CFS score. Regarding BNP, reportedly, elevated BNP (≥ 100 pg/ml is significantly associated with frailty in the general elderly population (OR 2.63; 95% confidence interval 1.61–4.32)^29^. Although the reasons for correlation between BNP levels and CFS scores in our study are not clear, it might be mediated by fluid overload and the presence of CVD, both of which are often seen in ESRD patients.

Patients of the frail group (mean CFS ≥ 5) had less planned dialysis initiation and longer hospitalization periods than patients of the non-frail group (mean CFS < 5). Although the reasons for these differences remain unclear, missing the appropriate timing to start dialysis can lead to an urgent start of dialysis and prolonged hospitalization, which undoubtedly imposes great physical and medical cost burdens on frail patients. This study revealed that that eGFR at dialysis initiation was significantly higher in the frail group, suggesting that eGFR based on serum creatinine might not reflect renal function in frail patients because of a loss of muscle mass, and that factors other than renal function (such as comorbidities) might have influenced the decision to initiate dialysis. Therefore, it might be necessary to consider not only objective clinical parameters but also frailty assessments based on clinical judgments to make decisions about dialysis initiation^30^.

The present study had several limitations. First, we might not have investigated or collected sufficient data of unknown factors affecting the relation between frailty and prognosis. Second, based on the nature of retrospective studies, potential influences of prognostic information related to the CFS assessment posed a concern. Therefore, the CFS evaluators made decisions based solely on information from the dialysis initiation period and were separated from the investigator for patient.

Third, because this study was conducted at a single tertiary hospital, it remains unclear whether the results were generalizable or not. Fourth, clinicians might misclassify the severity of CFS based on limited descriptions included in medical chart information. Based on information related to daily life including mobility, shopping, meal preparation, and bathing, CFS is a judgement-based tool to evaluate frailty^12^. At the time of admission to our hospital, this information had been obtained from the patient and family, and had been recorded carefully in the medical chart. We were able to evaluate CFS retrospectively based on this information. For some earlier studies, CFS was generated retrospectively from data of medical charts^31–33^. One of these studies has identified that agreement, accuracy, precision, and inter-rater reliability were similarly good when comparing retrospectively attained CFS scores from medical records and those assessed prospectively by interviewing patients^33^. It has been stated that CFS can be assessed without the patient having to visit a clinic in person^34^. Additionally, we tried to reduce the misclassification of patients by calculating the mean of CFS scores reported from two dialysis nurses.

Fifth, the change in CFS score before and after dialysis initiation was not investigated. Finally, cognitive function was not evaluated for this study, although recognition of patients with dementia might contribute to an adequate CFS classification. To address these limitations, a large and multicenter prospective cohort study with long-term follow-up must be undertaken as a future study.

In conclusion, results of this study demonstrated that frailty at the initiation of dialysis is related to death or hospitalization within 2 years. When used by nurses who are familiar with a patient's living and physical conditions, CFS might become an effective tool for producing prognoses for incident dialysis patients.

## Supplementary Information

Supplementary Tables.

Supplementary Figures.

## Data Availability

The data which support the findings of this study are available from the corresponding author, [Y.H.], upon reasonable request.
