# Supplementary material for: Clinical frailty assessment might be associated with mortality in incident dialysis patients
Source: Sci Rep. 2022 Oct 21;12:17651. doi: 10.1038/s41598-022-22483-8 (PMC9587224; doi:10.1038/s41598-022-22483-8)
Supplement: Supplementary file 1 — Supplementary Tables. [file 41598_2022_22483_MOESM1_ESM.pdf]

Supplementary materials

Supplementary Table 1. Results of univariate cox proportional hazards model analysis for 2-year mortality

**Variables**	**HR (95% CI)**	***p***
Age	1.03 (1.00–1.07)	.03
Male	2.41 (0.83–6.96)	.07
diabetic kidney disease as primary kidney disease	0.60 (0.26–1.38)	.22
planned initiation of dialysis	0.36 (0.16–0.78)	< .01
hemodialysis (vs. peritoneal dialysis)	3.84 (0.52–28.3)	.19
hospitalization period	1.01 (0.99–1.02)	.06
history of CVD	1.37 (0.64–2.91)	.42
systolic blood pressure	0.97 (0.96–0.99)	< .01
diastolic blood pressure	0.99 (0.96–1.01)	.31
body mass index	0.95 (0.87–1.02)	.18
mean CFS score	1.99 (1.53–2.58)	< .01
Hb	0.78 (0.62–0.99)	.04
Cr	0.69 (0.56–0.83)	< .01
BUN	1.01 (0.99–1.02)	.40
eGFR	1.29 (1.12–1.44)	< .01
corrected Ca	1.09 (0.65–1.82)	.74
IP	0.94 (0.71–1.20)	.65
TP	0.97 (0.55–1.76)	.93
Alb	0.37 (0.19–0.73)	< .01
CRP	1.15 (1.07–1.22)	< .01
T.chol	0.98 (0.97–0.99)	< .01
TG	0.997 (0.99–1.00)	.33
UA	1.16 (0.96–1.40)	.11
HbA1C	0.998 (0.60–1.54)	.99
BNP	1.00 (1.00–1.0004)	< .01

Supplementary Table 2. Results from multivariate cox proportional hazards model for 2-year mortality

**Variables**	Model 1		Mode1 2		Model 3	
	**OR (95% CI)**	***p***	**OR (95% CI)**	***p***	**OR (95% CI)**	***p***
Age	1.02 (0.99–1.05)	*.11*	1.02 (0.99–1.05)	*.09*	1.03 (0.99–1.06)	*.11*
mean CFS score	1.94 (1.40–2.66)	<*.01*	1.81 (1.38–2.39)	<*.01*	1.73 (1.28–2.31)	<*.01*
planned initiation of dialysis	0.92 (0.36–2.33)	*.86*				
systolic blood pressure			0.99 (0.97–1.01)	*.09*		
T chol					0.99 (0.98–0.998)	*.03*
BNP						
Cr						
Alb						
CRP						
**Variables**	Model 4		Mode1 5		Model 6	
	**OR (95% CI)**	***p***	**OR (95% CI)**	***p***	**OR (95% CI)**	***p***
Age	1.02 (0.99–1.05)	.20	1.01 (0.97–1.04)	.76	1.03 (0.99–1.06)	.06
mean CFS score	1.85 (1.37–2.48)	< .01	1.73 (1.29–2.31)	< .01	1.70 (1.26–2.30)	< .01
planned initiation of dialysis						
systolic blood pressure						
T chol						
BNP	1.00 (0.99–1.00)	.22				
Cr			0.78 (0.63–0.93)	< .01		
Alb			0.72 (0.35–1.48)	.37		
CRP					1.10 (0.997–1.18)	.057
