# Supplementary material for: Clinical frailty assessment might be associated with mortality in incident dialysis patients
Source: Sci Rep. 2022 Oct 21;12:17651. doi: 10.1038/s41598-022-22483-8 (PMC9587224; doi:10.1038/s41598-022-22483-8)
Supplement: Supplementary file 2 — Supplementary Figures. [file 41598_2022_22483_MOESM2_ESM.pdf]

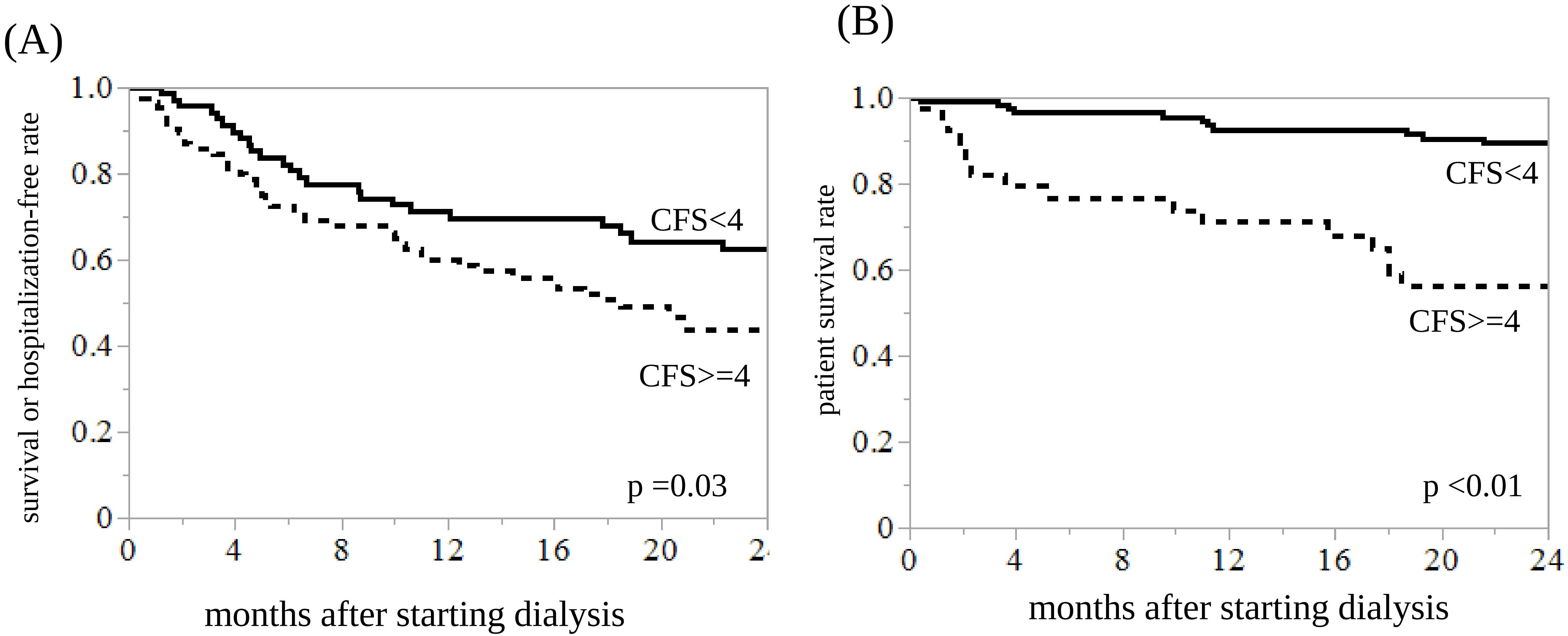

Supplementally Figure 1. Kaplan-Meier analysis for composite outcome (survival or hospitalization-free rate) (A) and survival rate (B) within 24 months after initiating dialysis.

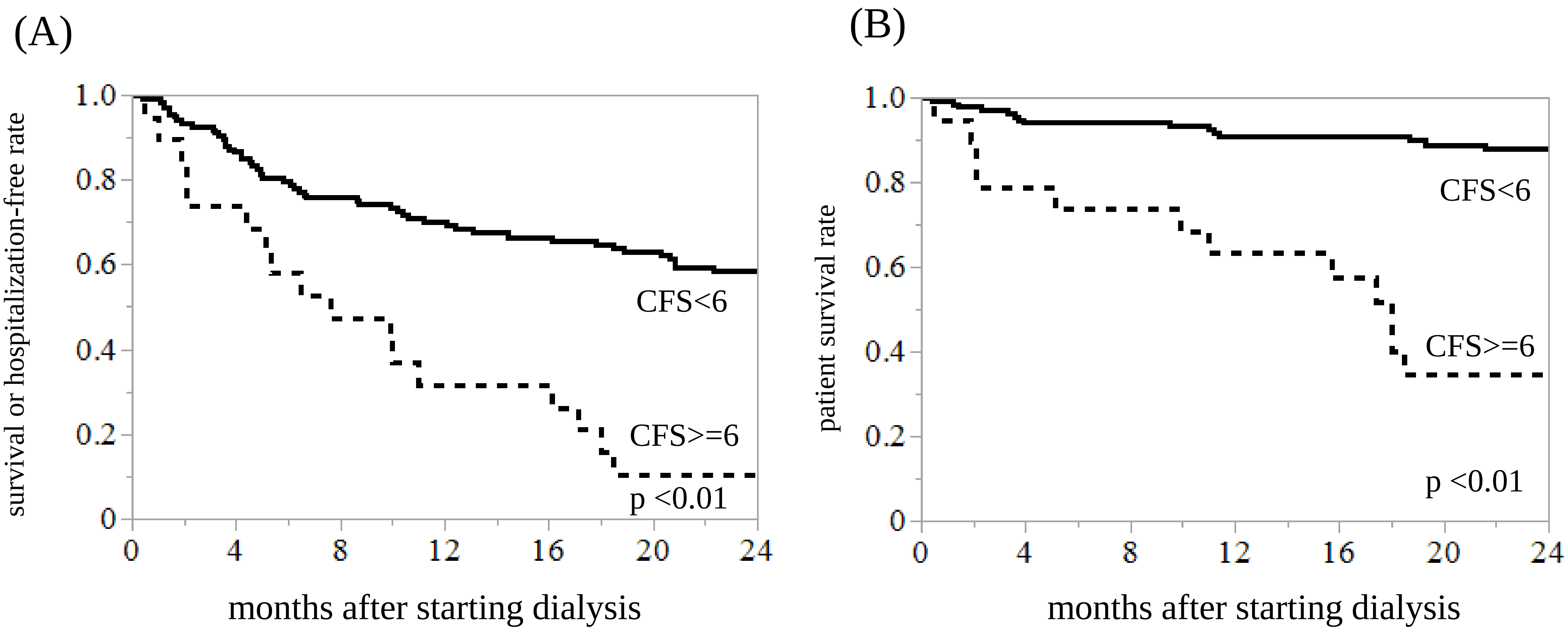

Supplementally Figure 2. Kaplan-Meier analysis for composite outcome (survival or hospitalization-free rate) (A) and survival rate (B) within 24 months after initiating dialysis.
